# The Effect of Alpha-Lipoic Acid on Mitochondrial Superoxide and Glucocorticoid-Induced Hypertension

**DOI:** 10.1155/2013/517045

**Published:** 2013-02-26

**Authors:** Sharon L. H. Ong, Harpreet Vohra, Yi Zhang, Matthew Sutton, Judith A. Whitworth

**Affiliations:** ^1^Department of Renal Medicine, St George Hospital, Kogarah, NSW 2217, Australia; ^2^Department of Medicine, University of New South Wales, Sydney, NSW 2052, Australia; ^3^John Curtin School of Medical Research, Australian National University, Canberra, ACT 0200, Australia; ^4^Cardiovascular Research Unit, The Canberra Hospital, Canberra, ACT 2605, Australia

## Abstract

*Aims*. To examine the effect of alpha-lipoic acid, an antioxidant with mitochondrial superoxide inhibitory properties, on adrenocorticotrophic hormone- (ACTH-HT) and dexamethasone-induced hypertensions (DEX-HT) in rats and if any antihypertensive effect is mediated via mitochondrial superoxide inhibition. *Methods*. In a prevention study, rats received ground food or alpha-lipoic-acid-laced food (10 mg/rat/day) for 15 nights. Saline, adrenocorticotrophic hormone (ACTH, 0.2 mg/kg/day), or dexamethasone (DEX, 10 **μ**g/rat/day) was injected subcutaneously from day 5 to day 11. In a reversal study, rats received alpha-lipoic-acid-laced food 4 days after commencement of saline or DEX. Tail-cuff systolic blood pressure (SBP) was measured second daily. Kidney mitochondrial superoxide was examined using (MitoSOX) Red (MitoSOX) via flow cytometry. *Results*. SBP was increased by ACTH (*P* < 0.0005) and DEX (*P* < 0.0005). Alpha-lipoic acid alone did not alter SBP. With alpha-lipoic acid pretreatment, SBP was increased by ACTH (*P*′ < 0.005) but not by DEX. Alpha-lipoic partially prevented ACTH-HT (*P*′ < 0.0005) and fully prevented DEX-HT (*P*′ < 0.0005) but failed to reverse DEX-HT. ACTH and DEX did not increase MitoSOX signal. In ACTH-hypertensive rats, high-dose alpha-lipoic acid (100 mg/rat/day) did not decrease SBP further but raised MitoSOX signal (*P* < 0.001), suggesting prooxidant activity. *Conclusion*. Glucocorticoid-induced hypertension in rats is prevented by alpha-lipoic acid via mechanisms other than mitochondrial superoxide reduction.

## 1. Introduction

Hypertension is a significant risk factor leading to cardiovascular and renal complications. One of the secondary causes of hypertension is glucocorticoid excess either due to endogenous overproduction or exogenous glucocorticoid administration [1]. The exact mechanism contributing to glucocorticoid-induced hypertension (GC-HT) has not been fully elucidated.

There is a body of evidence implicating oxidative stress in the pathogenesis of GC-HT [2]. We have previously shown that GC-HT in the rat was prevented and reversed by several antioxidants including tempol [3, 4], N-acetylcysteine [5, 6], and folate [7]. In rats, hypertension due to both adrenocorticotrophic hormone (ACTH) and dexamethasone (DEX) was accompanied by a raised plasma biomarker of lipid peroxidation, F_2_-isoprostane concentrations [3, 4, 7, 8]. In addition, increased lucigenin-enhanced chemiluminescence was also demonstrated in aortas of ACTH-hypertensive rats [4, 5].

Whilst there are several pathways contributing to the production of reactive oxygen species (ROS), not all of these are implicated in the pathogenesis of GC-HT. The NADPH oxidase [8, 9] but not xanthine oxidase [8, 10], cyclooxygenase [11], or eNOS in its uncoupled form [7, 12] plays a significant role in GC-HT.

The mitochondrion is another important source of ROS, primarily the superoxide radical and consequently its dismutation product, hydrogen peroxide, which can form highly reactive hydroxyl radicals. It consumes approximately 90% of the cell's oxygen and 1–4% of oxygen reacting with the mitochondrial respiratory chain is incompletely reduced to ROS [13, 14]. Complexes I (NADH dehydrogenase) and III (cytochrome *bc*_1_ complex) of the mitochondrial electron transport chain are generally accepted as the main source of ROS in the mitochondria. [15]. The role of mitochondrial ROS, on the other hand, has not been evaluated in the GC-HT model.

Alpha-lipoic acid, a naturally occurring short chain fatty acid, serves as an important cofactor for many enzyme complexes including mitochondrial respiratory enzymes. Exogenously administered alpha-lipoic acid has been shown to be an effective antioxidant. Urinary F_2_-isprostane concentration was significantly reduced after treatment with 600 mg/day alpha-lipoic acid orally for 2 months in humans [16]. Apart from this, alpha-lipoic acid has also been shown to improve mitochondrial function. A single dose of alpha-lipoic acid (100 mg/kg i.p.) resulted in improvement in mitochondrial function, determined by mitochondrial oxygen consumption and complex I, II, and IV activities, in endotoxemic rats [17]. Mitochondrial membrane potential is another indicator of mitochondrial health as it reflects the metabolic activity and integrity of mitochondrial membrane [18]. Alpha-lipoic acid supplementation (0.5% w/w) improved the average mitochondrial membrane potential in old rats hepatocytes to that of young rats [19].

Apart from improving mitochondrial function, treatment with alpha-lipoic acid prevented both hypertension and mitochondrial ROS overproduction due to hyperglycemia in rats and thus suggested a role for mitochondrial ROS in this form of experimental hypertension [20]. Mitochondrial superoxide dismutase deficiency has been shown to be linked with susceptibility to hypertension with aging and high salt intake in mouse [21]. The role of mitochondrial ROS overproduction in the development of GC-HT has not been established.

In this study, we evaluated the effect of alpha-lipoic acid on hypertension in rats and to determine if any antihypertensive effect is mediated via mitochondrial superoxide inhibition. The potential role of mitochondrial superoxide inhibition was examined in rat kidney mitochondria.

## 2. Materials and Methods

This study was approved by Animal Experimentation Ethics Committee of the Australian National University (Protocol no. J. HB. 20.05). Male Sprague-Dawley rats (body weight 200–300 g) were housed in individually ventilated plastic cages with a controlled temperature of between 21° to 23°C and a 12-hour light/dark cycle. The rats had free access to standard rat chow and tap water. Prior to any experimental procedure, the rats were acclimatized to their surroundings, food and water, handling, and tail-cuff sphygmomanometer.

### 2.1. Treatment Protocols

Alpha-lipoic acid powder (Sigma, St. Louis, USA) was administered by mixing in ground food and given to rats overnight (16–18 hours). Normal saline (0.9% NaCl, 0.1 mL/rat/day), DEX (10 *μ*g/rat/day) (David Bull Laboratories, Mulgrave, Victoria, Australia), and adrenocorticotrophic hormone (ACTH, Synacthen Depot, 0.2 mg/kg/day) (Novartis Pharmaceuticals, Sydney, NSW, Australia) were administered for 12 days from day T0 to T11 after 4 control days (C4–C1).

**Figure 1:**
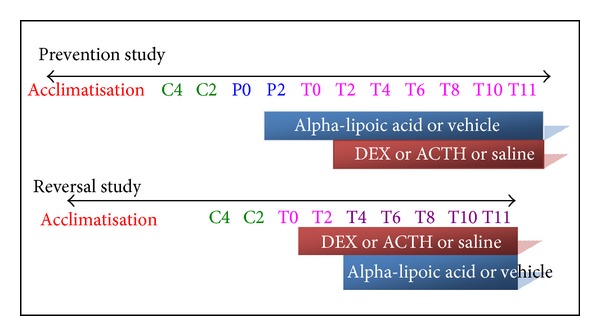
Timeline of experimental protocol.

The prefixes “P”, “T,” and “C” represent pretreatment, treatment, and control days, respectively. The timeline of the experimental protocol is summarized in Figure 1.

### 2.2. Animals

The rats were randomly divided into 9 treatment groups. The alpha-lipoic acid prevention study was performed in both DEX- and ACTH-HT. The reversal study was only performed in DEX-HT as alpha-lipoic acid completely prevented DEX-HT.

#### 2.2.1. Control Groups

Control rats were allocated 30 g of plain ground food per rat per night (16–18 hours) from P0. Pelleted food was given during the control days and daytime (from approximately 09:00 to 17:00 hours) from P0:

 group 1. Saline (*n* = 10),
 group 2. ACTH (*n* = 10),
 group 3. DEX (*n* = 10).

#### 2.2.2. Alpha-Lipoic Acid Prevention Studies

In the prevention studies, rats were pretreated with either alpha-lipoic acid (500 mg/kg in food) [20] in ground food overnight (16–18 hours) from P0, 4 days before commencement of saline, DEX, or ACTH injections. This dose (500 mg/kg in food) was effective in preventing hypertension and mitochondrial ROS overproduction due to hyperglycaemia in rats [20]. In another group of 4 rats, high dose alpha-lipoic acid (5 g/kg of ground food, starting at P0) was given prior to the commencement of ACTH injections from T0–T11:

 group 4. Alpha-lipoic acid + saline (*n* = 10),
 group 5. Alpha-lipoic acid + DEX (*n* = 10),
 group 6. Alpha-lipoic acid + ACTH (*n* = 10),
 group 7. Alpha-lipoic acid (high dose) + ACTH (*n* = 4).

#### 2.2.3. Alpha-Lipoic Acid Reversal Study

In the reversal studies, oral alpha-lipoic-acid-laced food was given 4 days after the subcutaneous injections (T4–T11):

 group 8. Saline + alpha-lipoic acid (*n* = 10),
 group 9. DEX + alpha-lipoic acid (*n* = 10).

### 2.3. Systolic BP and Body Weight Measurements

Systolic blood pressure (SBP) was measured by the same investigator second-daily using the tail-cuff blood pressure monitor (Narco Biosystems, Houston, USA) between 9:00–11:00 AM on conscious rats. The animals were placed in plastic restrainers on a heated plate (40°C) for 40 minutes. The animals were allowed to settle in the restrainers for 10–12 minutes before SBP readings were recorded. Several readings were obtained but the mean of 4 median readings, among which the difference was not greater than 10 mmHg, were taken as SBP. Body weight measurements were recorded on alternate days after the tail-cuff SBP measurements.

### 2.4. Measurement of Thymus Weight

On T11, the rats were sacrificed by exsanguination under isoflurane anaesthesia (4% induction and 2% maintenance) (Abbott Australasia, Kurnell, Australia) in the morning between 09:00 and 11:00 hours. Wet thymus weight, expressed relative to body weight (grams of thymus weight per 100 g body weight), was used as a marker of glucocorticoid activity.

### 2.5. Blood Glucose Determination

Blood was drawn via cardiac puncture. A drop of blood was placed on the glucometer strip (Precision Plus Blood Glucose Electrodes, Abbott Laboratories, MA, USA) and the blood glucose concentration was read on a glucometer (MediSense 2, Abbott Laboratories, Bedford, MA, USA).

### 2.6. Detection of Kidney Mitochondrial Superoxide

The rat kidney was resected, trimmed, immediately chilled, homogenised in phosphate buffered solution, and filtered to produce a single cell suspension. Cells were stained with Trypan blue (Fluka, Seelze, Germany) and counted. A total cell count of 2.0 × 10^6^ per sample was required for flow cytometry analysis with <20% dead cells in the cell suspension.

The cells were incubated with reconstituted MitoSOX Red dye (MitoSOX, Molecular Probes, Invitrogen, USA, 2 *μ*M) and 1,1′,3,3,3′,3′-hexamethylindodicarbocyanine iodide (DiIC_1_(5), Molecular Probes, Invitrogen, USA, 10 nM) for 10 minutes at 37°C prior to flow analysis. MitoSOX detects mitochondrial superoxide in live cells whilst DiIC_1_(5) determines the mitochondrial membrane potential. All samples were prepared in duplicate. Validation of this technique is described in *Supplementary Document 1 *in Supplementary Material available online at http://dx.doi.org/10.1155/2013/517045.

**Figure 2:**
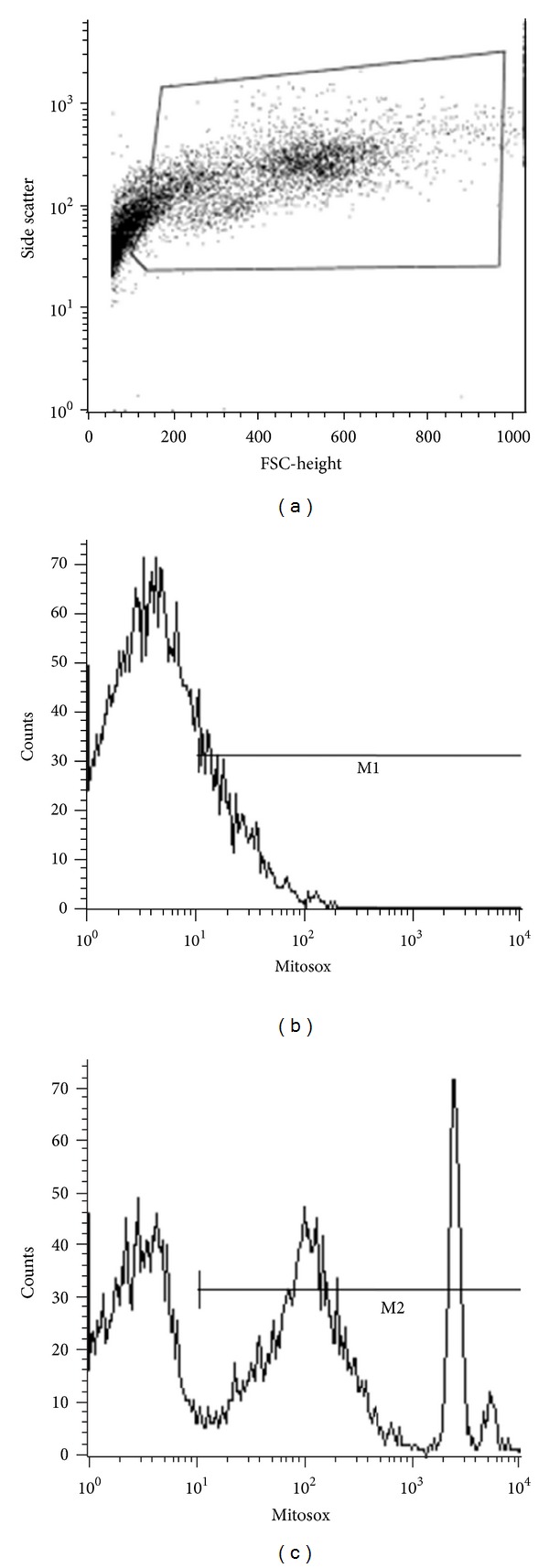
(a) Cells were gated to exclude debris. (b) Autofluorescence of unstained cells (control) in the marked region (M1) was recorded. (c) Fluorescence of cells treated with MitoSOX in the marked region (M2) was recorded. Net geometric MFI was calculated by subtracting the geometric MFI obtained in M2 region from M1 region. FSC = forward scatter.

Flow cytometric analysis was performed using a FACSort BD (Becton Dickinson, San Jose, USA) flow cytometer equipped with 488 nm Argon and 633 nm diode lasers. The approximate excitation and emission (Ex/Em) spectral peaks for MitoSOX are 510/580 and for DiIC_1_(5) are 638/658 nm. Excitation of the MitoSOX oxidation products was performed using the 488 nm laser, and DiIC_1_(5) was excited by the 633 nm laser. Cells were gated to exclude cell debris whilst covering a large side scatter to ensure the inclusion of all cell lines from the kidney (Figure 2(a)). The geometric mean fluorescence intensity (MFI) values of the samples were obtained by subtracting fluorescence of the stained specimen, obtained in the region marked M2 in Figure 2(c), with autofluorescence of unstained specimen obtained in the region marked M1 in Figure 2(b). 

### 2.7. *F*_2_-Isoprostane Assay

Blood was collected into chilled tubes containing 1 mg/mL ethylenediaminetetraacetate and 1 mg/mL reduced glutathione (Sigma, St. Louis, USA). Plasma was protected from oxidation by the addition of 0.2 mg/mL butylated hydroxytoluene (Sigma, St. Louis, USA) and samples stored at −70°C. Plasma F_2_-isoprostane concentrations were measured using electron-capture negative-ion gas chromatography-mass spectrometry as previously described [22].

### 2.8. Plasma Nitrate/Nitrite Assay

Plasma nitrate/nitrite was used as a marker of reactive nitrogen intermediates. It was determined by the reduction of nitrate to nitrite using the modified Griess colour reaction assay (Total Nitric Oxide Assay Kit, Pierce Endogen, Rockford, USA) as described previously [5].

### 2.9. Data and Statistical Analysis

Results were expressed as mean ± SEM. Results were analyzed by repeated measures analysis of variance with Greenhouse Geisser adjustment for multisample asphericity or unpaired *t*-test. The Ryan-Holm stepdown Bonferroni procedure was applied to raw *P* values to control the family-wise Type 1 error.

## 3. Results

### 3.1. Systolic Blood Pressure

DEX increased SBP from 115 ± 3 to 139 ± 4 mmHg (T0–T10, *P* < 0.005) and ACTH from 110 ± 3 to 133 ± 4 mmHg (T0–T10, *P* < 0.0005). Sham injection with sterile saline did not modify SBP (T0: 113 ± 2, T10: 119 ± 3 mmHg, *ns*). Between-group comparisons showed that SBPs in the groups receiving DEX and ACTH were significantly higher than those in the saline-treated group (*n* = 10 each, *P*′ < 0.001).

#### 3.1.1. Prevention Studies

**Figure 3:**
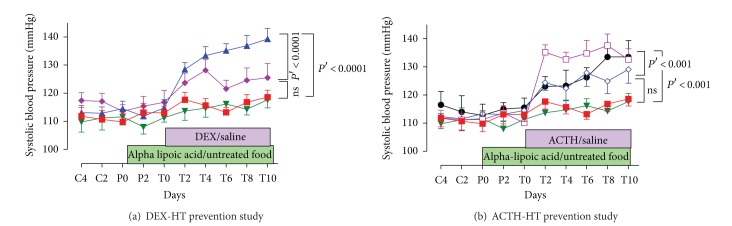
Tail-cuff systolic blood pressure in alpha-lipoic acid prevention studies. (a) DEX-HT, (b) ACTH-HT. ■ saline, *n* = 10; ▲ DEX, *n* = 10; □ ACTH, *n* = 10; *▼* alpha-lipoic acid + saline, *n* = 10; ♦ alpha-lipoic acid + DEX, *n* = 10, *◊* alpha-lipoic acid + ACTH, *n* = 10, ● high dose alpha-lipoic acid + ACTH, *n* = 4, data were presented as mean ± SEM.

There was no significant change in SBP in the alpha-lipoic acid + saline group (T0: 111 ± 2, T10: 118 ± 3 mmHg, *ns*). With alpha-lipoic acid pretreatment, DEX did not significantly increase SBP (T0: 117 ± 4, T10: 126 ± 5 mmHg, *ns*). However, there was a significant increase in SBP in ACTH-treated rats receiving alpha-lipoic acid pretreatment (T0: 115 ± 2 to T10: 129 ± 5 mmHg, *P* < 0.05). Despite this, there was a significant decrease in SBP in the alpha-lipoic acid + ACTH group (*P*′ < 0.001) when compared with the ACTH-only group, indicating partial prevention by alpha-lipoic acid. A tenfold increase in alpha-lipoic acid dose (100 mg/rat/day) did not decrease the SBP further. There was no difference in SBP between the alpha-lipoic acid + saline-treated group and the saline-only group. SBP of the alpha-lipoic acid + DEX-treated group was significantly lower than that of the DEX-only group (*P*′ < 0.001) (Figures 3(a) and 3(b)).

#### 3.1.2. Reversal Study

**Figure 4:**
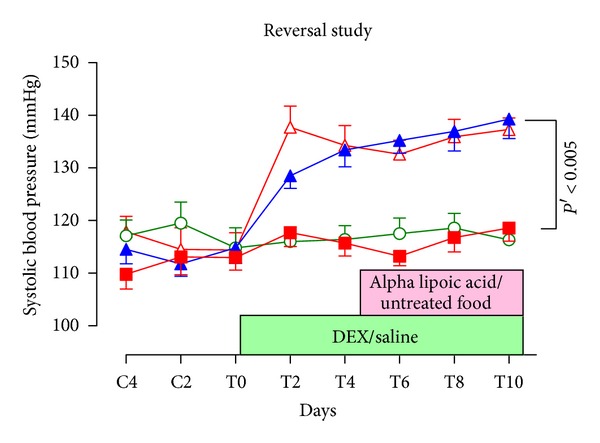
Tail-cuff systolic blood pressure in alpha-lipoic acid/DEX-HT reversal study. ■ saline, *n* = 10; ▲ DEX, *n* = 10; ○ saline + alpha-lipoic acid, *n* = 10; ∆ DEX + alpha-lipoic acid, *n* = 10, data were presented as mean ± SEM.

The increase in SBP due to DEX was not reversed by alpha-lipoic acid (10 mg/rat/day) treatment. Systolic BP on day T4, day 1 of alpha-lipoic acid treatment, was 134 ± 4 mmHg and on T10 was 137 ± 2 mmHg (*n* = 10, *ns*) in the DEX-treated group. As in the prevention study, alpha-lipoic acid did not alter SBP in the saline-treated group (T4: 116 ± 3 mmHg; T10: 116 ±2 mmHg, *n* = 10, *ns*) (Figure 4).

### 3.2. Body Weight

Body weight of saline-treated rats increased steadily from 270 ± 6 to 306 ± 7 g (T0–T10, *P* < 0.0005). DEX-treated rats did not gain significant body weight (T0: 272 ± 9, T10: 277 ± 7 g, T0–T10, *ns*) whilst ACTH treatment resulted in significant weight loss (from T0: 283 ± 11 to T10: 258 ± 8 g, *P* < 0.001). In these studies, the DEX- and ACTH-treated groups showed significantly lower body weights than the saline-treated group (*P*′ < 0.005).

#### 3.2.1. Prevention Studies

In the prevention studies, rats on saline + alpha-lipoic acid gained body weight steadily from T0: 271 ± 11 to T10: 295 ± 12 g (*n* = 10, *P* < 0.0005). Alpha-lipoic acid (10 mg/rat/day) pretreatment did not alter body weight in saline-, DEX-, or ACTH-treated rats significantly. However, rats on higher alpha-lipoic acid (100 mg/rat/day) dose and ACTH had significantly lower body weight compared with those on ACTH alone (*P*′ < 0.005).

#### 3.2.2. Reversal Study

In the reversal study, rats on DEX + alpha-lipoic acid failed to gain weight during the course of the study (from T4: 277 ± 10 to T10: 278 ± 8 g, *n* = 10, *ns*) whilst those on saline + alpha-lipoic acid gained weight progressively from 276 ± 10 g on day T4 to 296 ± 10 g on day T10 (*n* = 10, *P*′ < 0.0005). There was no significant difference in body weight between rats treated with DEX alone and rats treated with DEX + alpha-lipoic acid.

### 3.3. Thymus Weight

Thymus wet weight was significantly lower with DEX and ACTH treatments compared with saline (*P*′ < 0.005) regardless of the presence of alpha-lipoic acid treatment (*P*′ < 0.005) (Table 1).

### 3.4. Blood Glucose Concentration

Neither DEX nor ACTH altered the blood glucose concentrations in rats. Alpha-lipoic acid increased blood glucose concentration in saline- and DEX-treated rats in the reversal study. This effect was not observed in the DEX-HT and ACTH-HT prevention studies (Table 1).

### 3.5. Plasma *F*_2_-Isoprostane Concentration

The DEX-treated group had significantly higher plasma F_2_-isoprostane concentration compared with the saline-treated group (*P*′ < 0.01). Rats pretreated with alpha-lipoic acid before DEX treatment had significantly lower plasma F_2_-isoprostane concentration than those on DEX alone (*P*′ < 0.05). However, no significant difference was observed in the DEX-HT reversal study between the DEX and DEX + alpha-lipoic acid groups. There was no significant difference in plasma F_2_-isoprostane concentration in rats on ACTH treatment compared with those on saline treatment. Alpha-lipoic acid (both at low and high doses) did not alter plasma F_2_-isoprostane concentration in the ACTH-treated rats (Table 1).

### 3.6. Plasma Nitrate and Nitrite Concentration

There was no significant difference in plasma NO_*x*_ concentration in DEX- and ACTH-treated rats compared with saline-treated rats. Alpha-lipoic acid also did not alter the plasma NO_*x*_ concentrations in rats (Table 1).

### 3.7. Kidney MitoSOX Fluorescence

There was no significant difference in kidney MitoSOX fluorescence between DEX- or ACTH- and saline-treated rats. Rats treated with high dose of alpha-lipoic acid and ACTH had significantly higher kidney MitoSOX fluorescence compared with ACTH treatment alone (Table 1).

### 3.8. Kidney DiIC_1_(5) Fluorescence

DiIC_1_(5) mean geometric fluorescence was significantly higher in the groups which received alpha-lipoic acid (in the prevention and reversal studies) and saline compared to the saline-only group. There was no significant difference in kidney DiIC_1_(5) fluorescence between DEX- or ACTH- (both at low and higher dosages) and saline-treated rats (Table 1).

## 4. Discussion

In this study, DEX and ACTH administration in rats increased blood pressure without increasing the MitoSOX mean fluorescent intensity, suggesting a lack of effect of DEX and ACTH on kidney mitochondrial superoxide production in the kidney. The role of mitochondrial superoxide in GC-HT was further tested using the antioxidant alpha-lipoic acid which has been shown to inhibit mitochondrial superoxide production [20].

The antioxidant alpha-lipoic acid fully prevented DEX-HT but only partially prevented ACTH-HT. This partial effect on ACTH-HT was not due to inadequate dosing as a ten fold increase in the alpha-lipoic acid dose to 5 g/kg of ground food did not result in complete prevention of ACTH-HT. Alpha-lipoic acid was less effective in reversing established DEX-HT. Using the same dosage used in the prevention study, alpha-lipoic acid did not reverse DEX-HT. The blood pressure lower effects of alpha-lipoic acid did not correlate directly with mitochondrial superoxide availability as demonstrated by the kidney MitoSOX assay.

Even though alpha-lipoic acid has been shown to be effective in improving mitochondrial function and decreasing mitochondrial superoxide in rats [19, 20], its effect is not entirely specific to mitochondria. The antioxidant effect of alpha-lipoic acid, which is mediated by both the original alpha-lipoic acid compound and its metabolite dihydrolipoic acid, a more potent antioxidant, acts via a number of mechanisms such as metal chelation and direct ROS scavenging [23]. Dihydrolipoic acid can regenerate antioxidants such as glutathione and vitamin C [23]. Thus, the tail-cuff blood pressure results need to be interpreted in conjunction with other biochemical analysis of oxidative stress. In this study, we used plasma F_2_-isoprostane concentration, a product of lipid peroxidation as a marker of systemic oxidative stress, and kidney MitoSOX fluorescence signal as a marker of tissue mitochondrial superoxide availability.

Plasma F_2_-isoprostane concentration was significantly increased in DEX- but not ACTH-hypertensive rats. The latter finding was in contrast to previous findings that ACTH-HT was associated with raised plasma F_2_-isoprostane concentration, suggesting insufficient power to detect a difference [24]. Pooled F_2_-isoprostane concentration data from our laboratory showed that ACTH-hypertensive rats (*n* = 46) had a significant higher plasma F_2_-isoprostane concentration than the normotensive saline-treated rats (*n* = 48) [2]. The presence of a raised F_2_-isoprostane concentration in DEX-treated rats again confirmed the role of oxidative stress in its pathogenesis. Pretreatment with alpha-lipoic acid successfully prevented the increase of plasma F_2_-isoprostane concentration due to DEX. However, alpha-lipoic acid had no effect on the plasma F_2_-isoprostane in established DEX-HT.

Information on mitochondrial ROS has been based largely on studies performed on mechanically isolated mitochondrial preparations or submitochondrial particles. Mechanical isolation of mitochondria from their natural cellular environment through differential centrifugation can affect the bioenergetics of the electron transport chain. Nohl et al. argued that previous claims that mitochondrial ROS production is byproducts of cellular respirations may simply reflect bioenergetic artefacts consequent on mechanical mitochondrial isolation [25]. Some workers examined mitochondrial ROS in intact live cells to avoid this artefact though this remains challenging experimentally [26–28]. Whilst this is possible for detection of hydrogen peroxide which is membrane permeable and stable, the detection of mitochondrial superoxide in live cells becomes more problematic as it is heavily affected by mitochondrial superoxide dismutase, a tightly regulated superoxide scavenger within the mitochondria. Furthermore, their activity varies with the different cell lines. One of the techniques that is increasingly used is *in situ* evaluation of mitochondrial superoxide using a novel fluorescence dye, MitoSOX.

In this experiment, MitoSOX probe was used in flow cytometry to detect mitochondrial superoxide in live cells. The use of flow cytometry allows a large number of cells to be analysed rapidly. Simultaneous quantitative measurements of a number of parameters such as mitochondrial superoxide and membrane potential or other markers are also possible with this technique. In addition, cell debris can also be excluded from analysis. With this method, which only detects fluorescence associated with particles, lower MitoSOX concentrations can be used. Whilst 5 *μ*M is recommended for other fluorometric techniques, lower MitoSOX concentrations can be used in flow cytometry. In our validation experiments, we have shown that MitoSOX concentration of more than 5 *μ*M can result in reductions in mitochondrial membrane potentials and decrease the uptake and fluorescence of MitoSOX in this cell preparation. It is also important to do parallel assessment of mitochondrial membrane potential to ensure that cell treatments, including MitoSOX dye loading itself, do not depolarise the mitochondria in the cells as MitoSOX requires a membrane potential across the inner mitochondrial membrane to accumulate there. Otherwise, MitoSOX can be oxidised elsewhere in the cell and result in falsely positive signals for mitochondrial superoxide.

In this study, cells from the kidney were used for several reasons. Firstly, the kidney is a highly vascularised organ, rich in resistance blood vessels. Secondly, the kidney is relevant to GC-HT. We have previously shown that ACTH-HT, a model mainly mediated by cortisol, is associated with increased renal vascular resistance [29, 30]. Furthermore, the rise in SBP due to ACTH in rats was accompanied by reductions in iNOS and eNOS gene expression in the kidney [31].

The MitoSOX mean fluorescence intensity on kidney cells was not altered by DEX, ACTH, or alpha-lipoic acid (500 mg/kg of ground food). However, alpha-lipoic acid at 5 g/kg of ground food resulted in significantly higher MitoSOX mean fluorescence intensity. This was not associated with a decrease in mitochondrial membrane potential, a phenomenon known to result in a false increase in MitoSOX signals. It is possible that alpha-lipoic acid at a higher dose results in oxidative stress, most likely due to increased mitochondrial superoxide generation. The role of alpha-lipoic acid and its active metabolite, dihydrolipoic acid, as prooxidants has been reported in the literature [23, 32, 33]. In this study, the increase in mitochondrial superoxide production is unlikely to translate to systemic oxidative stress given the lack of change in the plasma F_2_-isoprostane concentration observed in this group, although the sample size for this group is very small and there may not have been significant power to detect a change. Another postulation is that superoxide generated within the mitochondria is not readily available to extramitochondrial structures. Superoxide, being a charged radical, does not permeate the mitochondrial membrane into the cytoplasm easily [34]. It leaves the mitochondria via voltage-dependent anion channels and more commonly, as membrane-permeable hydrogen peroxide following its dismutation by intermembrane Cu, Zn-superoxide dismutase [34].

The lower blood pressure readings observed in the alpha-lipoic acid + DEX and alpha-lipoic acid + ACTH groups in the prevention studies and DEX + alpha-lipoic acid in the reversal study were not due to inadequate dosing of DEX and ACTH. Similarly, the lower plasma F_2_-isoprostane in the alpha-lipoic acid + DEX-treated group compared to DEX-only group was not a reflection of insufficient DEX administration. The effectiveness of DEX and ACTH delivery was confirmed by significant lowering of thymus weight in all groups receiving DEX or ACTH due to thymocyte apoptosis and thymic involution induced by glucocorticoid excess [35, 36]. Alpha-lipoic acid has no effect on the GC-induced thymic involution.

In this study, blood glucose concentration was evaluated because hyperglycaemia has been shown to result in mitochondrial dysfunction [37] and is a common feature in humans exposed to excess glucocorticoid hormones. Fasting blood glucose was significantly higher in patients with active Cushing's syndrome than those in remission [38], and age- and sex-matched controls [39]. In this study, however, ACTH and DEX did not alter blood glucose concentration in rats, which are consistent with previous findings in rats [12]. Interestingly, alpha-lipoic acid significantly increased glucose concentration in the reversal study involving DEX-HT and its saline control. The fact that alpha-lipoic acid increased blood glucose concentration in DEX- and saline-treated rats in the reversal study but not in the prevention study suggests that duration of alpha-lipoic acid treatment is likely to play a role. However, there is no evidence in the literature implicating alpha-lipoic acid as a cause of hyperglycemia. Rather, alpha-lipoic acid at a same dose-prevented hyperglycemia in glucose-fed rats [40].

We have previously shown that plasma NO_*x*_ was decreased in DEX-HT [10, 24]. This decrease was not observed in the present studies on DEX- and ACTH-HT, with and without alpha-lipoic acid treatment. This may be influenced by decreased sensitivity of this assay in detecting very low plasma NO_*x*_ levels seen in all group including the control and DEX-treated groups. In this study, alpha-lipoic acid did not alter the plasma NO_*x*_ concentrations in rats treated with saline, DEX, or ACTH. The effect of alpha-lipoic acid on the saline-treated rats was consistent with the results seen in other studies where alpha-lipoic acid given to rats orally (20 mg/kg body weight [41] and 1 g/L drinking water [42]) did not alter plasma NO_*x*_ in normal controls. In these studies, alpha-lipoic acid prevented the rise in plasma NO_*x*_ induced by cyclosporine treatment [41] and biliary cirrhosis [42] in rats by the inhibition of renal iNOS and total hepatic NOS, respectively. In the present study, however, alpha-lipoic acid did not alter plasma NO_*x*_ in DEX- or ACTH-treated rats. This is probably because, unlike those studies, DEX and ACTH suppress eNOS and iNOS in rats [31, 43].

In summary, alpha-lipoic acid fully prevented DEX-HT and partially prevented ACTH-HT without altering the kidney mitochondrial superoxide availability. This suggests that GC-HT is prevented (and DEX-HT, partially reversed) by alpha-lipoic acid through a mechanism other than mitochondrial superoxide reduction.

The differences observed between DEX- and ACTH-HT, once again, depict the different pathophysiological mechanisms contributing to the two hypertensive models.

## Supplementary Material

This supplementary material provides additional information on some of the validation experiments performed in this study. The minimum MitoSOX concentration required to provide an optimal fluorescence signal in rat kidney cells was determined in the first validation study. MitoSOX concentration of 2 *μ*M was identified as the lowest optimal concentration. MitoSOX geometric mean fluorescence intensity was increased in rat kidney cells pre-treated in Antimycin A (5 *μ*M, positive control) and decreased in cells pre-treated with Tiron (50 mM, negative control).

## Figures and Tables

**Table 1:** Biological measurements for alpha-lipoic acid studies.

Groups	Thymus (mg/100 g body weight)	Blood glucose (mmol/L)	Plasma NO_*x*_ (*μ*M)	Plasma F_2_-isoprostane (nmol/L)	MitoSOX (geometric MFI)	DiIC_1_ (5) (geometric MFI)
Saline	127 ± 7.2	11 ± 0.3	9.3 ± 1.2	4.7 ± 0.3	198 ± 15	73 ± 8
DEX	48 ± 4.1*	9 ± 0.4*	12.3 ± 0.9	7.1 ± 0.6*	223 ± 21	107 ± 18
ACTH	48 ± 3.2*	11 ± 0.6	8.2 ± 0.8	5.4 ± 0.4	196 ± 7	110 ± 13
LA + saline p	140 ± 15.8	11 ± 0.6	13.0 ± 1.5	4.9 ± 0.3	201 ± 11	184 ± 12*
LA + DEX p	50 ± 2.0^*⋆*^	10 ± 0.3	11.2 ± 1.0	5.7 ± 0.2^†^	220 ± 18	131 ± 8
LA + ACTH p	53 ± 3.7^*⋆*^	10 ± 0.5	13.1 ± 3.4	5.4 ± 0.3	217 ± 13	152 ± 20
HD LA + ACTH p	53 ± 2.1	13 ± 1.1	8.1 ± 1.0	4.6 ± 0.6	273 ± 10^Δ^	125 ± 6
Saline + LA r	133 ± 10.0	12 ± 0.5*	12.7 ± 2.3	5.4 ± 0.2	166 ± 18	161 ± 10*
DEX + LA r	52 ± 3.1^#^	12 ± 0.8^†^	10.9 ± 1.5	5.6 ± 0.5	178 ± 20	134 ± 12

LA: alpha-lipoic acid, HD LA: high dose alpha-lipoic acid, p: prevention, and r: reversal. *P′ < 0.05 versus saline, ^⋆^ *P*′ < 0.005 versus LA + saline prevention, ^#^ *P*′ < 0.01 versus saline + LA reversal, ^†^ *P*′ < 0.05 versus DEX, and ^Δ^ *P*′ < 0.0005 versus ACTH. Data presented as mean ± SEM.

## References

[1] Whitworth JA, Mangos GJ, Kelly JJ (2000). Cushing, cortisol, and cardiovascular disease. *Hypertension*.

[2] Ong SLH, Zhang Y, Whitworth JA (2008). Reactive oxygen species and glucocorticoid-induced hypertension. *Clinical and Experimental Pharmacology and Physiology*.

[3] Zhang Y, Jang R, Mori TA (2003). The anti-oxidant Tempol reverses and partially prevents adrenocorticotrophic hormone-induced hypertension in the rat. *Journal of Hypertension*.

[4] Zhang Y, Croft KD, Mori TA, Schyvens CG, McKenzie KUS, Whitworth JA (2004). The antioxidant tempol prevents and partially reverses dexamethasone-induced hypertension in the rat. *American Journal of Hypertension*.

[5] Mondo CK, Zhang Y, De Macedo Possamai V (2006). *N*-acetylcysteine antagonizes the development but does not reverse ACTH-induced hypertension in the rat. *Clinical and Experimental Hypertension*.

[6] Krug S, Zhang Y, Mori TA (2008). *N*-acetylcysteine prevents but does not reverse dexamethasone-induced hypertension. *Clinical and Experimental Pharmacology and Physiology*.

[7] Miao Y, Zhang Y, Lim PS (2007). Acid prevents and partially reverses glucocorticoid-induced hypertension in the rat. *American Journal of Hypertension*.

[8] Zhang Y, Chan MMK, Andrews MC (2005). Apocynin but not allopurinol prevents and reverses adrenocorticotropic hormone-induced hypertension in the rat. *American Journal of Hypertension*.

[9] Hu L, Zhang Y, Lim PS (2006). Apocynin but not L-arginine prevents and reverses dexamethasone-induced hypertension in the rat. *American Journal of Hypertension*.

[10] Ong SLH, Vickers JJ, Zhang Y (2007). Role of xanthine oxidase in dexamethasone-induced hypertension in rats. *Clinical and Experimental Pharmacology and Physiology*.

[11] Zhang Y, Miao Y, Whitworth JA (2007). Aspirin prevents and partially reverses adrenocorticotropic hormone-induced hypertension in the rat. *American Journal of Hypertension*.

[12] Zhang Y, Pang T, Earl J (2004). Role of tetrahydrobiopterin in adrenocorticotropic hormone-induced hypertension in the rat. *Clinical and Experimental Hypertension*.

[13] Richter C (1988). Do mitochondrial DNA fragments promote cancer and aging?. *FEBS Letters*.

[14] Lenaz G (1998). Role of mitochondria in oxidative stress and ageing. *Biochimica et Biophysica Acta*.

[15] Muller FL, Liu Y, Van Remmen H (2004). Complex III releases superoxide to both sides of the inner mitochondrial membrane. *Journal of Biological Chemistry*.

[16] Marangon K, Devaraj S, Tirosh O, Packer L, Jialal I (1999). Comparison of the effect of *α*-lipoic acid and *α*-tocopherol supplementation on measures of oxidative stress. *Free Radical Biology and Medicine*.

[17] Vanasco V, Cimolai MC, Evelson P, Alvarez S (2008). The oxidative stress and the mitochondrial dysfunction caused by endotoxemia are prevented by *α*-lipoic acid. *Free Radical Research*.

[18] Distelmaier F, Koopman WJH, Testa ER (2008). Life cell quantification of mitochondrial membrane potential at the single organelle level. *Cytometry A*.

[19] Hagen TM, Ingersoll RT, Lykkesfeldt J (1999). (R)-*α*-lipoic acid-supplemented old rats have improved mitochondrial function, decreased oxidative damage, and increased metabolic rate. *The FASEB Journal*.

[20] El Midaoui A, Elimadi A, Wu L, Haddad PS, De Champlain J (2003). Lipoic acid prevents hypertension, hyperglycemia, and the increase in heart mitochondrial superoxide production. *American Journal of Hypertension*.

[21] Rodriguez-Iturbe B, Sepassi L, Quiroz Y, Ni Z, Vaziri ND (2007). Association of mitochondrial SOD deficiency with salt-sensitive hypertension and accelerated renal senescence. *Journal of Applied Physiology*.

[22] Mori TA, Croft KD, Puddey IB, Beilin LJ (1999). An improved method for the measurement of urinary and plasma F_2_- isoprostanes using gas chromatography-mass spectrometry. *Analytical Biochemistry*.

[23] Biewenga GP, Haenen GRMM, Bast A (1997). The pharmacology of the antioxidant: lipoic acid. *General Pharmacology*.

[24] Zhang YI, Jason HY, Janine JA (2009). The role of 20-hydroxyeicosatetraenoic acid in glucocorticoid-induced hypertension. *Journal of Hypertension*.

[25] Nohl H, Gille L, Staniek K (2005). Intracellular generation of reactive oxygen species by mitochondria. *Biochemical Pharmacology*.

[26] Duranteau J, Chandel NS, Kulisz A, Shao Z, Schumacker PT (1998). Intracellular signaling by reactive oxygen species during hypoxia in cardiomyocytes. *Journal of Biological Chemistry*.

[27] Vanden Hoek TL, Shao Z, Li C, Schumacker PT, Becker LB (1997). Mitochondrial electron transport can become a significant source of oxidative injury in cardiomyocytes. *Journal of Molecular and Cellular Cardiology*.

[28] Dawson TL, Gores GJ, Nieminen AL, Herman B, Lemasters JJ (1993). Mitochondria as a source of reactive oxygen species during reductive stress in rat hepatocytes. *American Journal of Physiology*.

[29] Wen C, Fraser T, Li M, Turner SW, Whitworth JA (1999). Haemodynamic mechanisms of corticotropin (ACTH)-induced hypertension in the rat. *Journal of Hypertension*.

[30] Wen C, Fraser T, Li M, Whitworth JA (1998). Hemodynamic profile of corticotropin-induced hypertension in the rat. *Journal of Hypertension*.

[31] Lou YK, Wen C, Li M (2001). Decreased renal expression of nitric oxide synthase isoforms in adrenocorticotropin-induced and corticosterone-induced hypertension. *Hypertension*.

[32] Çakatay U (2006). Pro-oxidant actions of *α*-lipoic acid and dihydrolipoic acid. *Medical Hypotheses*.

[33] Moini H, Packer L, Saris NEL (2002). Antioxidant and prooxidant activities of *α*-lipoic acid and dihydrolipoic acid. *Toxicology and Applied Pharmacology*.

[34] Han D, Antunes F, Canali R, Rettori D, Cadenas E (2003). Voltage-dependent anion channels control the release of the superoxide anion from mitochondria to cytosol. *Journal of Biological Chemistry*.

[35] Sun XM, Dinsdale D, Snowden RT, Cohen GM, Skilleter DN (1992). Characterization of apoptosis in thymocytes isolated from dexamethasone-treated rats. *Biochemical Pharmacology*.

[36] Zavitsanou K, Nguyen V, Greguric I, Chapman J, Ballantyne P, Katsifis A (2007). Detection of apoptotic cell death in the thymus of dexamethasone treated rats using [^123^I]Annexin V and in situ oligonucleotide ligation. *Journal of Molecular Histology*.

[37] Munusamy S, MacMillan-Crow LA (2009). Mitochondrial superoxide plays a crucial role in the development of mitochondrial dysfunction during high glucose exposure in rat renal proximal tubular cells. *Free Radical Biology and Medicine*.

[38] Terzolo M, Allasino B, Bosio S (2004). Hyperhomocysteinemia in patients with Cushing's syndrome. *Journal of Clinical Endocrinology and Metabolism*.

[39] Faggiano A, Pivonello R, Spiezia S (2003). Cardiovascular risk factors and common carotid artery caliber and stiffness in patients with Cushing's disease during active disease and 1 year after disease remission. *Journal of Clinical Endocrinology and Metabolism*.

[40] El Midaoui A, Wu R, De Champlain J (2002). Prevention of hypertension, hyperglycemia and vascular oxidative stress by aspirin treatment in chronically glucose-fed rats. *Journal of Hypertension*.

[41] Amudha G, Josephine A, Sudhahar V, Varalakshmi P (2007). Protective effect of lipoic acid on oxidative and peroxidative damage in cyclosporine A-induced renal toxicity. *International Immunopharmacology*.

[42] Marley R (1999). Lipoic acid prevents development of the hyperdynamic circulation in anesthetized rats with biliary cirrhosis. *Hepatology*.

[43] Wallerath T, Witte K, Schäfer SC (1999). Down-regulation of the expression of endothelial NO synthase is likely to contribute to qlucocorticoid-mediated hypertension. *Proceedings of the National Academy of Sciences of the United States of America*.

