# Supplementary material for: The Effect of Alpha-Lipoic Acid on Mitochondrial Superoxide and Glucocorticoid-Induced Hypertension
Source: Oxid Med Cell Longev. 2013 Feb 26;2013:517045. doi: 10.1155/2013/517045 (PMC3600316; doi:10.1155/2013/517045)
Supplement: Supplementary file 1 — This supplementary material provides additional information on some of the validation experiments performed in this study. The minimum MitoSOX concentration required to provide an optimal fluorescence signal in rat kidney cells was determined in the first validation study. MitoSOX concentration of 2 μM was identified as the lowest optimal concentration. MitoSOX geometric mean fluorescence intensity was increased in rat kidney cells pre-treated in Antimycin A (5 μM, positive control) and decreased in cells pre-treated with Tiron (50 mM, negative control). [file 517045.f1.zip › 517045.f1.pdf]

Flow cytometry analysis validation

Prior to the above analysis, a series of experiments were conducted to validate the mitochondrial superoxide assay using MitoSOX via flow cytometry. These experiments were performed on cells obtained from kidneys of 6 untreated male Sprague-Dawley rats using the method described above. Flow cytometric acquisition and analysis were performed using the method described previously.

To evaluate the concentration of MitoSOX which will result in optimal signal, kidney cells were stained with MitoSOX at concentrations 1, 2, 3, 4, 5, 6, 7, 8, 9, 10µM using the method described above. Concentrations up to 3 µM resulted in a steady increase in MitoSOX geometric MFI. This signal plateaued at concentrations between 3-6 µM and declined at concentrations > 6 µM (Figure 5). To minimise risk of cytotoxicity initiated by MitoSOX, which might in turn result in cell and mitochondrial damage, a concentration 1 µM below the concentration that produced the maximal geometric MFI (ie 2 µM) was chosen.

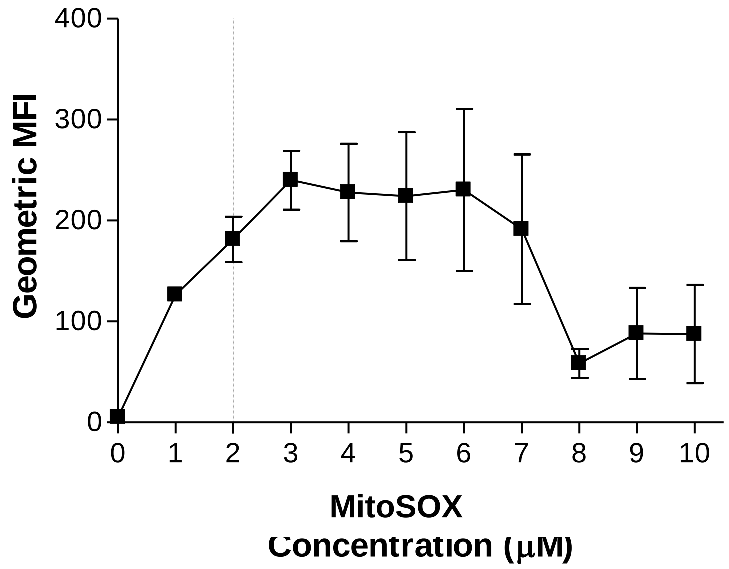

Figure 5: MitoSOX titration curve, n = 4.

Antimycin A (Sigma-Aldrich, St Louis, USA), a mitochondrial complex IV inhibitor known to induced mitochondrial superoxide production, was used in establishing a positive control. The kidney cells were treated with Antimycin A (5µM) prior to staining with MitoSOX and $DiIC_1(5)$. The geometric MFI for both dyes were evaluated using the flow cytometric analysis method described above. Cells pre-treated with 5 µM Antimycin A had significantly higher MitoSOX fluorescence (694 ± 105, n = 6) compared with untreated cells (control, 213 ± 27, n = 6, $P < 0.005$) (Figure 6). Antimycin A at 5 µM did not significantly reduce mitochondrial membrane potential as indicated by $DiIC_1(5)$ geometric MFI (control: 136 ± 49 and Antimycin A: 72 ± 12, n = 6 each, *ns*) (Figure 7).
