# Supplementary material for: The Effect of Alpha-Lipoic Acid on Mitochondrial Superoxide and Glucocorticoid-Induced Hypertension
Source: Oxid Med Cell Longev. 2013 Feb 26;2013:517045. doi: 10.1155/2013/517045 (PMC3600316; doi:10.1155/2013/517045)
Supplement: Supplementary file 1 — This supplementary material provides additional information on some of the validation experiments performed in this study. The minimum MitoSOX concentration required to provide an optimal fluorescence signal in rat kidney cells was determined in the first validation study. MitoSOX concentration of 2 μM was identified as the lowest optimal concentration. MitoSOX geometric mean fluorescence intensity was increased in rat kidney cells pre-treated in Antimycin A (5 μM, positive control) and decreased in cells pre-treated with Tiron (50 mM, negative control). [file 517045.f1.zip › 517045.f3.pdf]

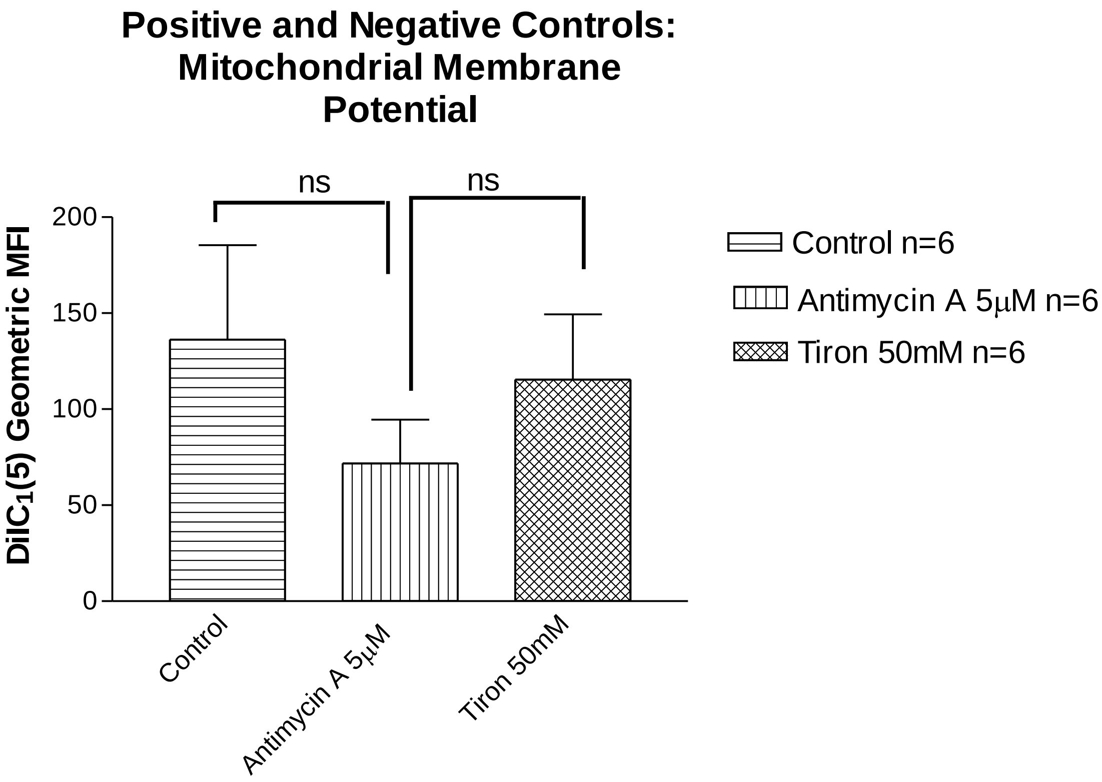

Figure 7: Mitochondrial membrane potential for the positive and negative controls established using Antimycin A and Tiron, respectively.

Tiron (Fluka, Steinheim, Germany), a superoxide mimetic agent, was used to lower superoxide in the mitochondria. Tiron solution (50mM) was added to the cells before the addition of mitoSOX and $DiIC_1(5)$ stains. Tiron at 50 mM resulted in significant reduction in MitoSOX geometric MFI (37 ± 6, n = 6) compared to controls (213 ± 27, n = 6, $P < 0.0005$) (Figure 6). At this concentration, Tiron did not significantly alter mitochondrial membrane potential as represented by $DiIC_1(5)$ geometric MFI (control: 136 ± 49 and Tiron: 115 ± 34, n = 6 each, *ns*) (Figure 7).
